# Prediction of Parameters of Equivalent Sum Rough Surfaces

**DOI:** 10.3390/ma13214898

**Published:** 2020-10-31

**Authors:** Pawel Pawlus, Rafal Reizer, Wieslaw Zelasko

**Affiliations:** 1 Faculty of Mechanical Engineering and Aeronautics, Rzeszow University of Technology, Powstancow Warszawy 8 Street, 35-959 Rzeszow, Poland; 2 College of Natural Science, University of Rzeszow, Pigonia Street 1, 35-310 Rzeszow, Poland; rreizer@ur.edu.pl; 3 Faculty of Mechanics and Technology, Rzeszow University of Technology, Kwiatkowskiego Street 4, 37-450 Stalowa Wola, Poland; w.zelasko@prz.edu.pl

**Keywords:** contact mechanics, equivalent sum rough surface, surface topography, parameters

## Abstract

In statistical models, the contact of two surfaces is typically replaced by the contact of a smooth, flat, and an equivalent rough sum surface. For the sum surface, the zeroth, second, and fourth moments of the power spectral density m_0_, m_2_, and m_4_ respectively, are the sum of spectral moments of two contacted surfaces. In this work, the selected parameters of the sum surfaces were predicted when the parameters of individual surfaces are known. During parameters selection, it was found that the pair of parameters: Sp/Sz (the emptiness coefficient) and Sq/Sa, better described the shape of the probability ordinate distribution of the analyzed textures than the frequently applied pair: the skewness Ssk and the kurtosis Sku. It was found that the RMS height Sq and the RMS slope Sdq were predicted with very high accuracy. The accuracy of prediction of the average summit curvature Ssc, the areal density of summits Sds, and parameters characterizing the shape of the ordinate distribution Sp/Sz and Sq/Sa was also good (the maximum relative errors were typically smaller than 10%).

## 1. Introduction

All surfaces are rough. Surface topography characterization is important during studies of various phenomena such as friction, wear, and contact resistance. Surface topography is of prime importance in problems of contact mechanics [1]. The contact of random isotropic Gaussian surfaces was typically studied. From among various models of random surface description, the model proposed by Nayak [2] was frequently applied. This model is based on earlier works of Longuet-Higgins [3,4], who described ocean surfaces. However, many surfaces are anisotropic. Nayak’s model was extended to anisotropic surfaces [5].

According to Nayak, the statistical parameters characterizing an isotropic Gaussian surface can be expressed in terms of spectral moments of profiles. The m_0_ moment is the profile variance, the m_2_ moment is the profile mean square slope, and the m_4_ moment is the mean square curvature of the surface profile. Greenwood and Tripp [6] found that the contact of two surfaces can be replaced by the contact of the equivalent sum rough surface and the smooth flat surface. The elastic modulus of the equivalent surface can be obtained from the following equation:

(1) $$E^{\prime }={\left(\frac{1-\nu_{1^{2}}}{E_{1}}+\frac{1-\nu_{2^{2}}}{E_{2}}\right)}^{-1}$$

where *E_i_* and *ν_i_* (*i* = 1, 2) are Young’s moduli and Poisson’s ratios of the two contacting elements [6]. 

**Figure 1:**
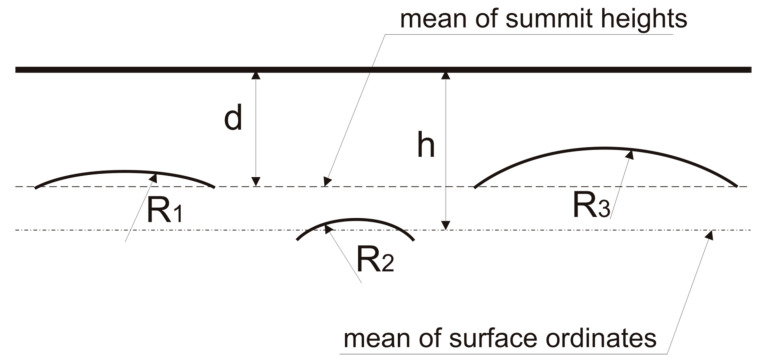
Scheme of the contact of rough surfaces.

Figure 1 shows a scheme of the contact of two rough surfaces. A separation of surfaces measured from the summits mean plane is called d, while h means a separation based on surface heights. R_1_, R_2_, and R_3_ are radii of the summits in contact. One can see that radius of summits, density, and height of summits are important parameters in contact mechanics.

To simplify the problem of the contact of two rough surfaces, researchers typically considered an equivalent surface in the contact with a smooth plane [7,8,9]. They found that the contact of two surfaces was negligibly different from the contact of a smooth flat and the equivalent sum rough surface. The m_0_ moment is the profile variance, the m_2_ moment is the profile mean square slope, and the m_4_ moment is the mean square curvature of the surface profile. These moments are substantial in contact of two surfaces. This simplification can be used not only in statistical models of the elastic contact [7,9], but also in statistical models of the elastic–plastic contact [10,11,12,13]. The contact parameters such as the number of contacting asperities, the real area of contact, and the contact load, for any given separation between the equivalent sum rough surface and a smooth flat, can be also calculated by summing the contributions of all the contacting asperities using the summit identification model [14,15,16]. Combining two rough surfaces onto one equivalent rough surface and a smooth plane can also be helpful in deterministic contact models considering the Boussinesq problem [17,18,19]. In all cases, the ordinates of the equivalent sum rough surface should be the sum of the ordinates of two contacted surfaces. For the equivalent rough surface, the spectral moments are the sum of the spectral moments of two individual surfaces [20]. The parameters important in rough contact mechanics, such as the average curvature of summits, Ssc, and the areal summit density, can be computed from the spectral moments [2,21]:

(2) $$\mathrm{Ssc}=\frac{8m_{4}^{\frac{1}{2}}}{3\pi^{\frac{1}{2}}}$$

(3) $$\mathrm{Sds}=\frac{1}{6\pi \sqrt{3}}\left(\frac{m_{4}}{m_{2}}\right)$$

These equations were proved only for surfaces of Gaussian ordinate distribution.

However, the distribution of surface height is often different from Gaussian [20,22,23]. Specifically, two-process surfaces characterized by negative values of the skewness, Ssk, and high values of the kurtosis, Sku, are of high functional importance [16,24,25,26]. Two-process textures have traces of two processes (machining or wear). The contact of deterministic surfaces is also possible.

In this work, the selected parameters of the equivalent sum rough surfaces will be predicted when the parameters of each surface are known.

## 2. Analyzed Textures

Surface textures were measured using a white light interferometer Talysurf CCI Lite (produced by Taylor Hobson Ltd., Leicester, UK). A vertical resolution was 0.01 nm. The measured area 3.3 × 3.3 mm^2^ and contained 1024 × 1024 points. Spikes and isolated deep and narrow valleys were eliminated by truncation of the height corresponding to material ratios of 0.01–99.99%. Before calculations of parameters, flat surfaces were leveled, while forms of the curved surfaces were removed using the polynomials of the second degree. Digital filtration was not used. We tried to analyze surfaces of various types: isotropic, anisotropic, random, deterministic, or mixed, of symmetric and non-symmetric ordinate distribution, one-process, and two-process. Therefore, surfaces after vapor blasting, polishing, lapping, milling, one- and two-process honing (plateau honing), and vapor blasting followed by lapping were measured. In most cases, the measured surfaces were machined using typical techniques. 

**Figure 2:**
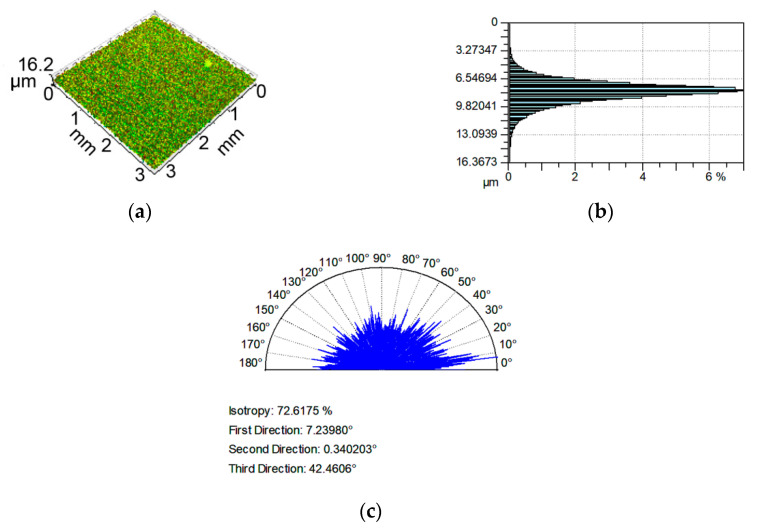
Isometric view (**a**), ordinate distribution (**b**), and directionality plot (**c**) of surface texture after vapor blasting.

**Figure 3:**
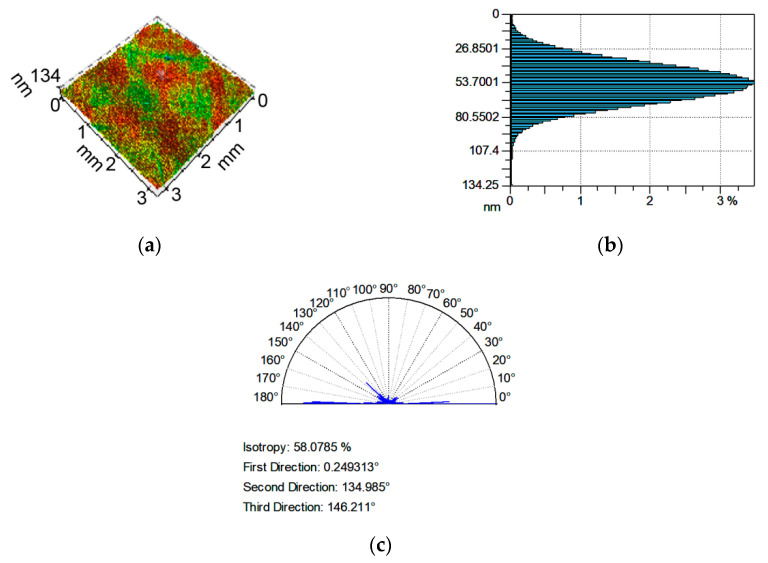
Isometric view (**a**), ordinate distribution (**b**), and directionality plot (**c**) of surface texture after polishing.

**Figure 4:**
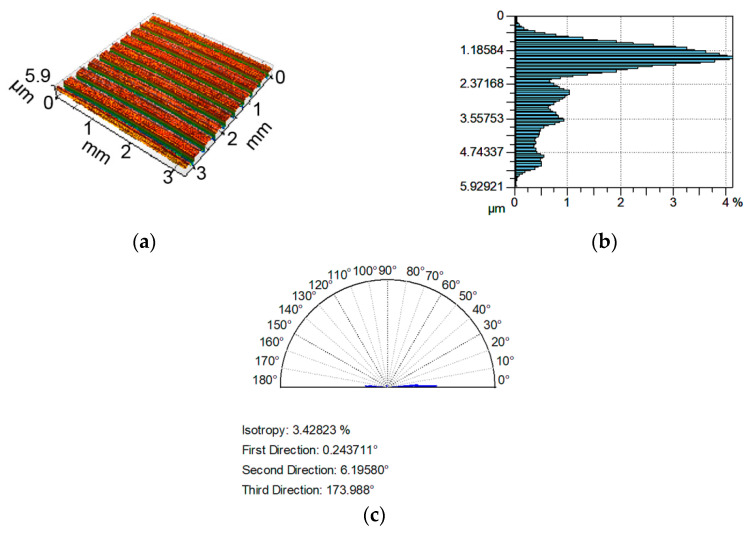
Isometric view (**a**), ordinate distribution (**b**), and directionality plot (**c**) of surface texture after milling.

**Figure 5:**
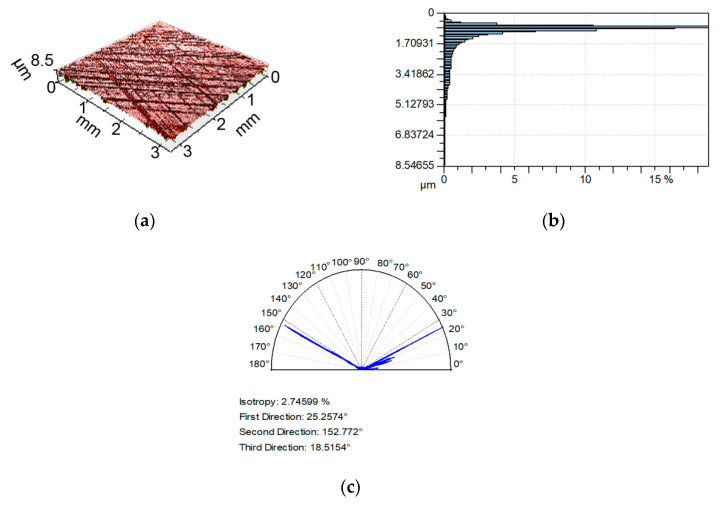
Isometric view (**a**), ordinate distribution (**b**), and directionality plot (**c**) of surface texture after plateau honing.

Examples of analyzed textures are shown in Figure 2, Figure 3, Figure 4 and Figure 5. They present isometric views, ordinate distributions, and directionality plots of selected surfaces. Surface after vapor blasting shown in Figure 2 is an isotropic texture of comparatively big height and Gaussian ordinate distribution. Surface presented in Figure 3 is a smooth random mixed Gaussian texture after polishing. Figure 4 shows deterministic texture after milling of an asymmetric ordinate distribution. These surfaces represent one-process textures. Figure 5 presents anisotropic cross-hatched random two-process texture after plateau honing of asymmetric ordinate distribution.

Forty textures were measured. In order to obtain equivalent rough surfaces, the ordinate distributions of measured surfaces were summed using TalyMap software: 50 equivalent sum surfaces were obtained. Parameters of individual surfaces and parameters of the sum surface were also calculated using the TalyMap software.

## 3. Selection of Surface Texture Parameters

The Sq parameter is a root mean square height of the surface.

(4) $$\mathrm{Sq}=\sqrt{\frac{1}{A}\iint_{A}z^{2}\left(x,y\right)\mathrm{dxdy}}$$

Where:

A—area,

z—surface height in position x, y,

x, y—lengths in perpendicular directions.

Sq is a statistical parameter of comparatively small sensitivity on the measurement errors [27]. It is frequently used in measurements of optical surfaces. This parameter is related to the standard deviation of asperity heights, which is frequently used in contact mechanics [9,10,11,12,13].

Sa is arithmetical mean surface height.

(5) $$\mathrm{Sa}=\frac{1}{A}\iint_{A}\left|z\left(x,y\right)\right|\mathrm{dxdy}$$

The Sa parameter is also non-sensitive to measurement errors. It is frequently used in machining.

Sdq, which is a root mean square gradient, is the hybrid parameter. Surface slope is used in assessing surface ability to the plastic deformation [28,29,30]. Slope is also related to friction, hydrodynamic lubrication, reflectance [31], and strength of adhesive joints [32]. The Sdq parameter is computed using the following formula:

(6) $$\mathrm{Sdq}=\sqrt{\frac{1}{A}\iint_{A}\left[{\left(\frac{\partial z\left(x,y\right)}{\partial x}\right)}^{2}+{\left(\frac{\partial z\left(x,y\right)}{\partial y}\right)}^{2}\right]\mathrm{dxdy}}$$

The Sq and Sdq parameters are included in ISO 25178-2 (Geometrical Product Specifications (GPS)—Surface texture: Areal—Part 2: Terms, definitions and surface texture parameters) standard [33].

The other parameters: the average summit curvature, Ssc, and areal density of summits, Sds, are related to summits, therefore they are important in contact characteristics [9,10,11,12,13]. The Ssc parameter enables us to know the mean form of the peaks: according to the mean value of the curvature of the surface at these points. The Ssc and Sds parameters are presented in an older standard, EUR15178N [34]. In calculations of the Sds and Ssc parameters, a point is considered as a summit if its ordinate is higher than those of its 8 neighbors. This definition is in accordance with References [5,14,15]. Therefore, these parameters are useful in the analysis of the contact of rough surfaces. In the new ISO 25178 standard, the Spc parameter (arithmetical mean peak curvature) replaces the Ssc parameter and the Spd parameter (density of peaks) replaces the Sds parameter. The Spc and Spd parameters are calculated in the same way as Ssc and Sds parameters but take into account only those significant summits that remain after a discrimination by segmentation [35]. Therefore, the value of the Spd parameter is smaller than that of the Sds parameter. However, because the Spc and Spd parameters do not consider all the peaks existed on a surface, the Ssc and Sds parameters will be analyzed.

The spatial surface properties are characterized by Sal and Str parameters. The Sal, the autocorrelation length, is the horizontal distance, at which the autocorrelation function slowly decays to 0.2 value. A high value of this parameter indicates that surface has mainly high wavelengths. The Str, the texture-aspect ratio, is the ratio of the shortest to the highest correlation lengths. This parameter has values between 0 (anisotropic surface) and 1 (isotropic surface).

The parameters characterizing the shape of the surface topography ordinate distribution are also important in contact mechanics [20,22,23]. Typically, this shape is characterized by the skewness, Ssk, and the kurtosis, Sku [36,37,38].

(7) $$\mathrm{Ssk}=\frac{1}{{\mathrm{Sq}}^{3}}\left[\frac{1}{A}\iint_{A}z^{3}\left(x,y\right)\mathrm{dxdy}\right]$$

(8) $$\mathrm{Sku}=\frac{1}{{\mathrm{Sq}}^{4}}\left[\frac{1}{A}\iint_{A}z^{4}\left(x,y\right)\mathrm{dxdy}\right]$$

The skewness, Ssk, characterizes the symmetry of the surface texture. The value of the skewness depends on if the material is above (negative skewed) or below (positive skewed) the mean plane. The kurtosis, Sku, describes the sharpness of the surface ordinate distribution. When Sku < 3, the surface has relative few high peaks and deep valleys, and when Sku > 3, the surface has many high peaks and deep valleys [39]. However, due to the large exponent used, the parameters Ssk and Sku are sensitive to presences of spikes as well as narrow and deep valleys. In addition, for two-process surfaces [16,24,25,26], these parameters are interrelated. Sp is the maximum peak height and Sz is the maximum surface height. The Rp, Rz, Rq, Ra, Rsk, and Rku are profile equivalents of the parameters Sp, Sz, Sq, Sa, Ssk, and Sku, respectively. It was found [40] that the pair of ratios Rp/Rz, Rq/Ra can replace the pair Rsk, Rku in description of the probability distribution of the roughness profile. The recommended parameters can be used for characterization of both one-process and two-process surfaces. These parameters are, in contrast to the pair (Rsk, Rku), statistically independent. The Rq/Ra ratio is much more stable on surface and has smaller sensitivity to the measurement errors than the kurtosis Rku.

**Figure 6:**
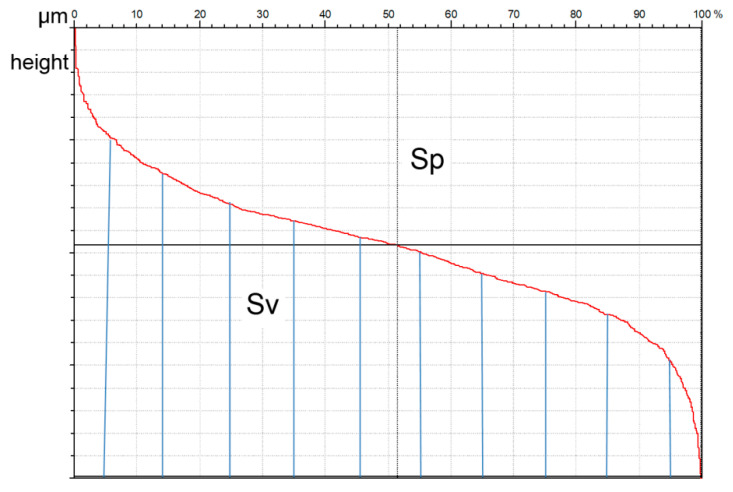
Graphical interpretation of Sp and Sv parameters.

The Sv parameter is the maximum valley depth (Sz = Sp + Sv). Figure 6 presents the graphical interpretation of the Sp and Sv parameters. Sv describes the area under, while Sp the area above the material ratio curve. This curve presents the cumulative distribution of surface ordinates. The Sv parameter presents material, while Sp presents void (emptiness). The Rp/Rz or Sp/Sz ratios are called the emptiness coefficient. One can believe that when this ratio is smaller, the wear intensity is also lower [41].

In other to analyze the possibility of using Sp/Sz and Sq/Sa parameters for description of the ordinate distribution of diversified textures, the correlation and regression analysis of forty surfaces was carried out. The linear coefficient of correlation was also used in previous studies [42,43,44,45]. Table 1 lists the values of the linear coefficient of correlation among the analyzed parameters.

**Figure 7:**
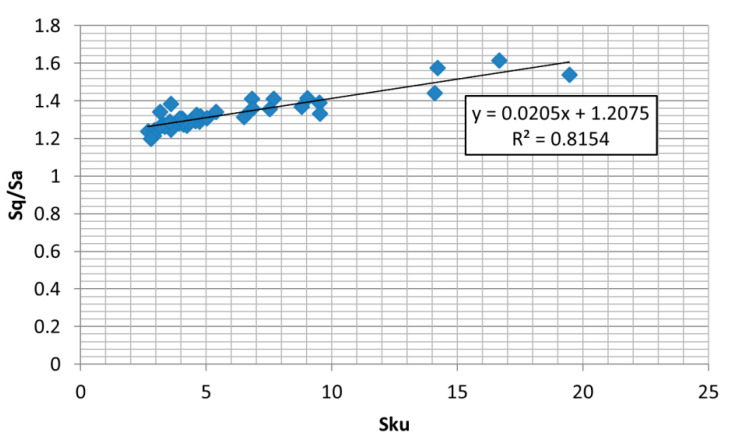
Dependence between parameters Sku and Sq/Sa.

It was assumed that when the absolute value of the coefficient of correlation was higher than 0.7, the parameters were strongly correlated (the coefficient of determination was larger than 0.5). One can see from the analysis of Table 1 that the Ssk parameter is strongly correlated with the Sku parameter (r = −0.77), therefore they cannot be used for description of the ordinate distributions of the analyzed textures. In contrast, the suggested parameters Sq/Sa and Sp/Sz are statistically independent (r = −0.45). The emptiness coefficient describes similar surface property as the skewness Ssk (r = 0.71). The Sq/Sa parameter is correlated with both Ssk (r = 0.91) and Sku (r = −0.82), however, the relation of Sq/Sa to the skewness, Ssk, is the strongest (Figure 7). On the basis of the presented analysis and due to larger stability and smaller sensitivity to measurement errors of the Sq/Sa ratio compared to the kurtosis, Sku, the pair Sq/Sa and Sp/St is recommended for description of the ordinate distribution of diversified surface textures (one- and two-process, random and periodic, isotropic and anisotropic).

The parameters describing the shape of the ordinate distribution, Ssk, Sku, Sp/Sz, and Sq/Sa, were not correlated with other analyzed parameters. The Sq parameter was proportional to Sdq (r = 0.75) and Sds (r = −0.72). The Sdq parameter was strongly correlated with the parameters Str (r = 0.79), Sds (−0.76), and Ssc (r = 0.97).

## 4. Methods of Parameters Prediction

The Sq parameter is related to m_0_ spectral moment, which is the profile variance. The Sdq parameter should be related to m_2_ moment, which is a square of the profile RMS slope. The Ssc parameter can be related to m_4_ moment which is the mean square curvature of the surface profile. For surface of Gaussian ordinate distribution, Equation (2) presents the dependence between m_4_ profile spectral moment and the average curvature of summits. Because the spectral moment of the equivalent rough surface is the sum of spectral moments of both surfaces, these parameters of equivalent surfaces were predicted using the following formulae:

(9) $$Sq_{sum}=\sqrt{Sq_{1}{}^{2}+Sq_{2}{}^{2}}$$

(10) $$Sdq_{sum}=\sqrt{Sdq_{1}{}^{2}+Sdq_{2}{}^{2}}$$

(11) $$Ssc_{sum}=\sqrt{Ssc_{1}{}^{2}+Ssc_{2}{}^{2}}$$

It would be difficult to predict the areal summit density, Sds, using Equation (3), because summit density depends on both m_2_ and m_4_ spectral moments. Furthermore, this formula was proven only for a selected type of textures.

Therefore, the Sds and other analyzed parameters (Sq/Sa, Sp/Sz, Ssk, Sku, Sal, and Str) were predicted as the weighted averages of them and of the Sq parameter of both textures:

(12) $$P_{sum}=\frac{Sq_{1}P_{1}+Sq_{2}P_{2}}{Sq_{1}+Sq_{2}}$$

where *P*_1_ is a parameter of the first surface, *P*_2_ is a parameter of the second surface, while *P_sum_* is predicted parameter of the sum surface. Equation (12) is an original conception of the authors of this paper.

## 5. Results of Parameters Prediction and Discussion

Table 2 presents the results of this study.

**Figure 8:**
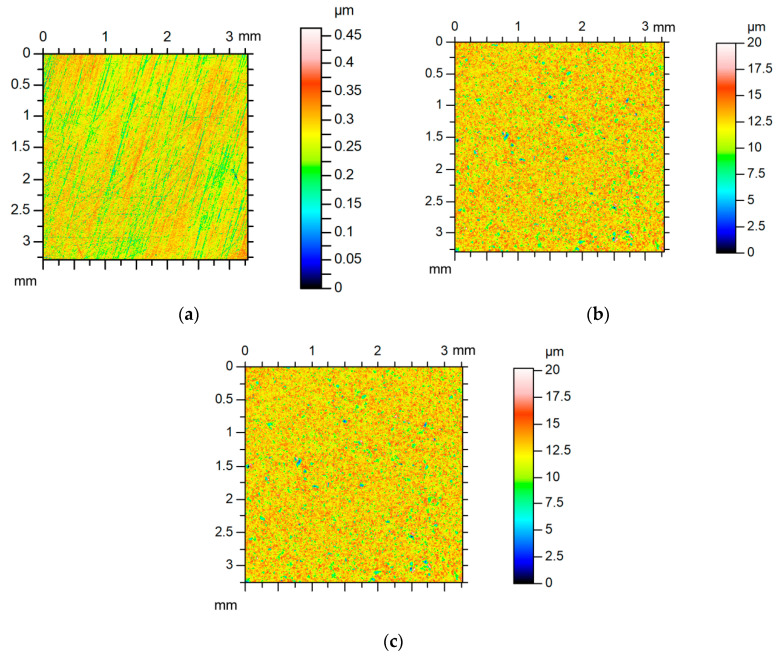
Contour plots of surface A (**a**), of surface B (**b**), and of equivalent sum surface (**c**).

Some examples of surfaces’ summations are given below. Surface A after lapping was a smooth anisotropic one-process random structure of a little asymmetrical ordinated distribution. Surface B was an isotropic one-process random texture of Gaussian ordinate distribution characterized by high roughness. Due to different roughness heights, the equivalent rough sum surface was similar to surface B (Figure 8). Table 3 presents parameters of surface A, of surface B, of the sum surface, and predicted parameters of the sum surface. Due to the large difference between amplitudes of two surfaces, the errors of parameters’ predictions were rather small. The largest errors were found for the Sal (about 8%) and the Str parameters (about 6%).

**Figure 9:**
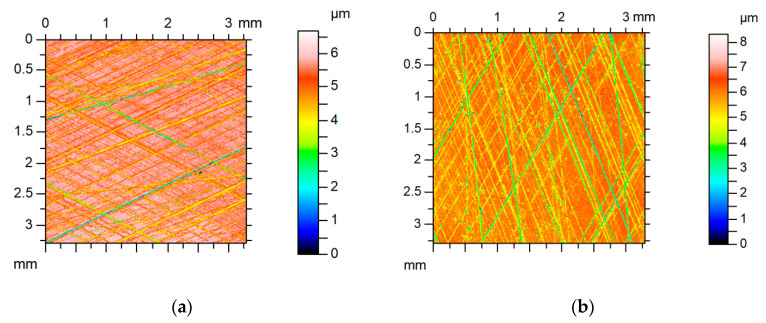

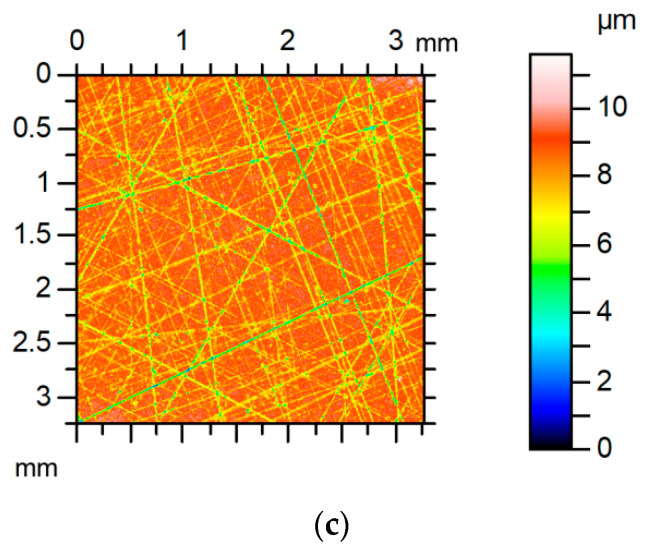
Contour plots of surface C (**a**), of surface D (**b**), and of equivalent sum surface (**c**).

**Figure 10:**
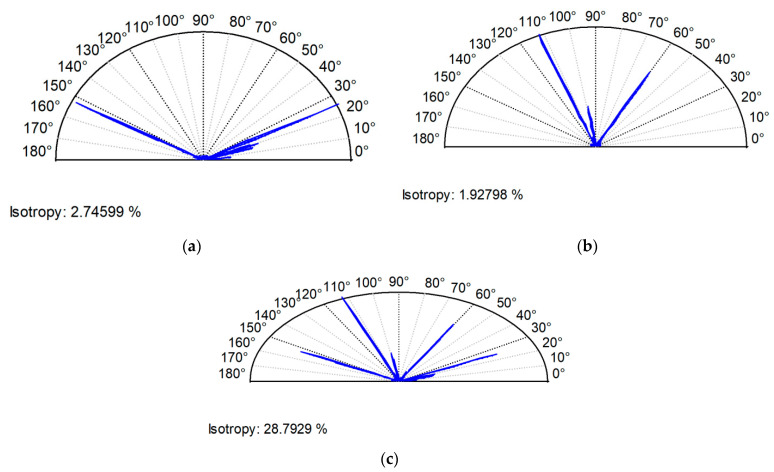
Directionality plots of surface C (**a**), of surface D (**b**), and of equivalent sum surface (**c**).

For the contact between both two-process surfaces C and D of similar characteristics, the predicted skewness, Ssk, was underestimated (Table 4). This was caused by the presence of more deep valleys on the sum surface compared to individual surfaces in the contact (Figure 9). The skewness, Ssk, is more negative for smaller number of deep valleys. The error of skewness prediction was large (40%). This is related to the large error of the kurtosis, Sku, prediction (near 50%). An increase in the number of valleys during creation of the sum surface caused a large increase in the texture aspect ratio, Str (Figure 10), and in the autocorrelation length, Sal. The error of the Str parameter prediction was about 90%, while the error of the Sal parameter expectation was 30%. The errors of other parameters’ predictions were smaller, up to 5% (Sds and Sq/Sa).

Comparatively low errors of parameters’ predictions were obtained for the contact of anisotropic surface E and isotropic surface F. Both surfaces were characterized by similar surface heights (the values of the Sq parameter were higher than 2). Surface E from cylinder liner was a cross-hatched structure obtained after one-process honing, however, surface B was isotropic texture after vapor blasting. Table 5 presents parameters of these surfaces, of the sum surface, and of predicted parameters of the sum surface.

**Figure 11:**
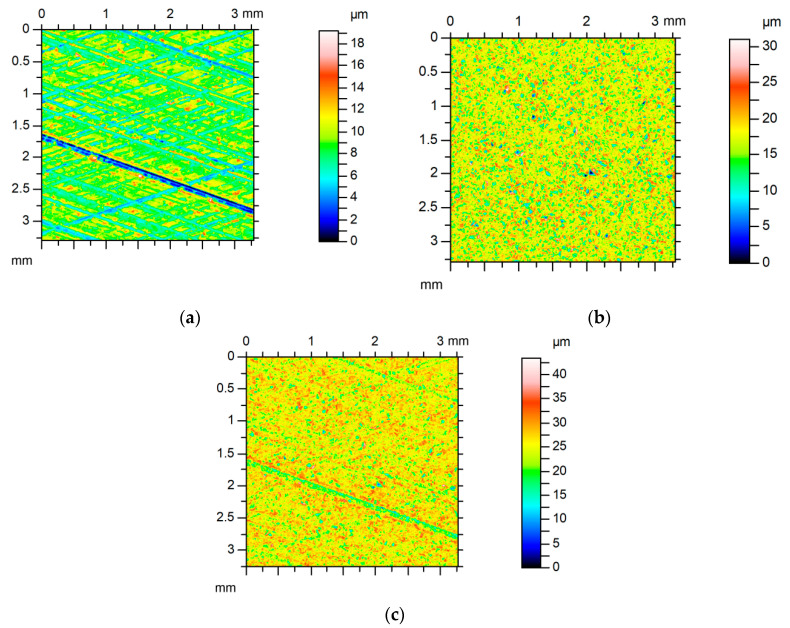
Contour plots of surface E (**a**), of surface F (**b**), and of equivalent sum surface (**c**).

**Figure 12:**
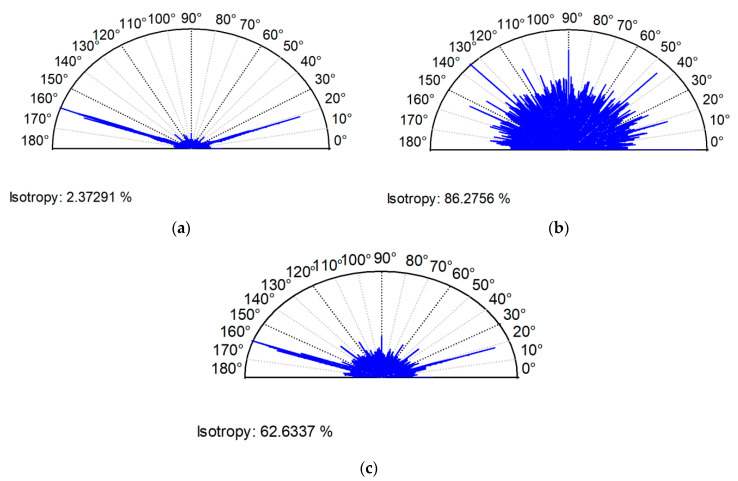
Directionality plots of surface E (**a**), of surface F (**b**), and of equivalent sum surface (**c**).

The errors of the Sq, Sdq, Ssc, Sds, Sp/Sz, Sq/Sa, and Sal parameters’ predictions were smaller than 4.5%. Two surfaces were characterized by slightly negative values of skewness. Similar to surfaces analyzed earlier, the skewness of the sum surface was higher than predicted (the relative error was about 30%), but the kurtosis was smaller (the error was about 16%) than predicted. The parameter Str of the equivalent sum surface was higher by about 17% than the predicted parameter. Addition of anisotropic honed surface to isotropic surface after vapor blasting caused a decrease in an isotropy ratio compared to isotropic texture. However, the change was lower than predicted. Figure 11 shows the contour plots and Figure 12 shows the directionality plots of surfaces E and F and of the sum surface. 

**Figure 13:**
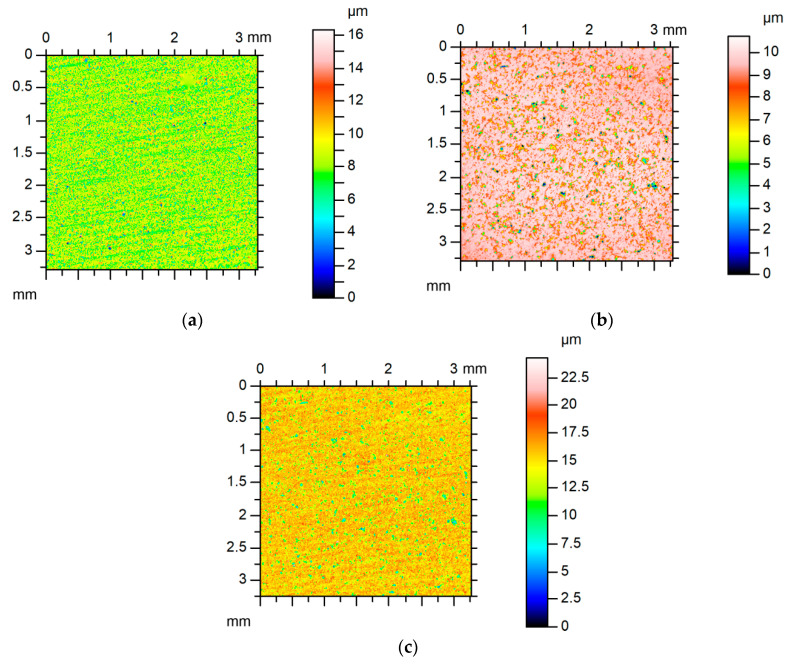
Contour plots of surface G (**a**), of surface H (**b**), and of equivalent sum surface (**c**).

**Figure 14:**
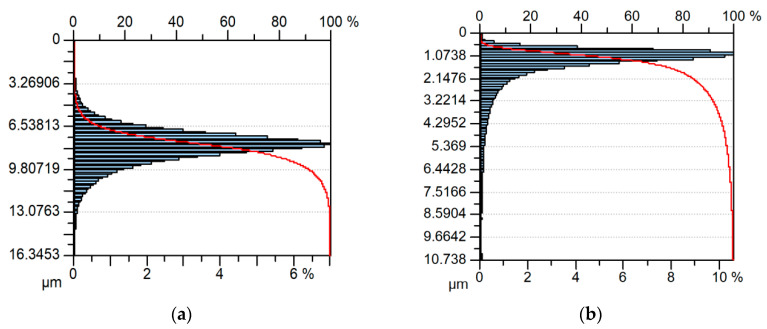

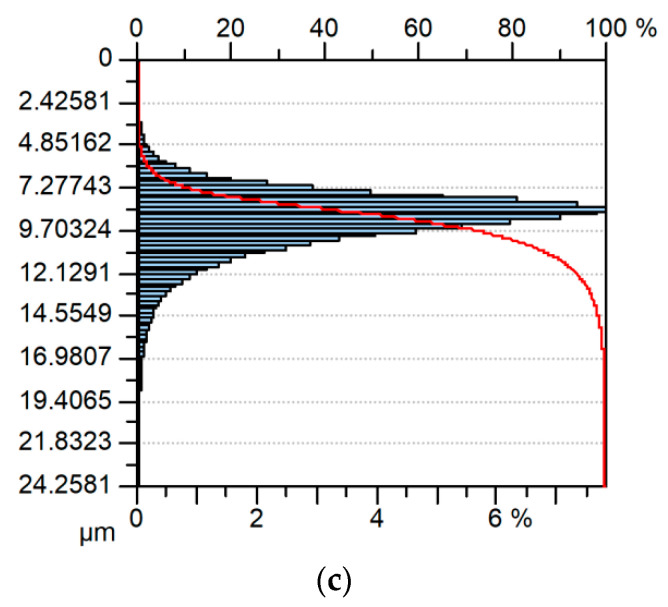
Material ratio curves and ordinate distributions of surface G (**a**), of surface H (**b**), and of equivalent sum surface (**c**).

When isotropic one-process surface G was added to isotropic two-process surface H (Figure 13), the errors of predictions of most parameters (Sq, Sdq, Sds, Str, and Sal) were comparatively low (smaller than 4.5%)—Table 6. However, relative differences among real and predicted parameters describing shapes of the ordinate distributions of analyzed textures were larger (Figure 14). The predicted Ssk parameter was underestimated (the error was near 50%), while the Sku parameter was overestimated. The errors of parameters Sp/Sz and Sq/Sa predictions were smaller and amounted to 11% and 6%, respectively.

The square root of m_0_ spectral moment is the Pq parameter of the profile or the Sq parameter of isotropic surface topography. When anisotropic one-directional surface texture is analyzed (for example, after grinding or milling), its Sq parameter is equal (or similar) to the mean value of the Pq parameter of the profile measured across the lay (main surface wavelength). In the perpendicular direction (along the lay), the mean value of the Pq parameter is smaller than the Sq parameter of areal surface texture. According to Equation (9), the square of the Sq parameter of the sum surface should be the sum of squares of the Sq parameters of both surfaces in the contact. This assumption was confirmed. For 50 sum surfaces, the maximum error was 0.99%, and the average error was 0.31%.

The analysis of surface slope is more complicated. The square root of m_2_ moment is the Pdq parameter, which is the RMS slope of the profile. Of course, this slope can be obtained using various methods (based on 2, 3, or 6 neighboring points). The method based on 2 points gave typically more correct value than the other methods [46].

For profiles of normal ordinate distribution, the rms. slope Pdq is approximately the average slope Pda magnified by 1.25. Areal (3D) slope is much larger than profile (2D) slope. For isotropic surfaces of Gaussian ordinate distribution, the following relation between the average slope of profile Pda and the areal topography slope, Sda, exists, according to Nayak [2]:

(13) $$\mathrm{Sda}=\frac{\pi }{2}\mathrm{Pda}$$

RMS slopes of profile and surface topography are related by similar dependence. For anisotropic surface, spectral moments of second and fourth order should be equal to the square root of the product from moments obtained in two perpendicular directions [5]. However, the Sdq parameter is calculated in a different manner—see Equation (6). When the ratio of larger to smaller average slopes in 2 perpendicular directions was higher than 3.5, the areal slope was similar to the larger profile slope. For two-process surfaces, the ratio of the RMS slope to the average slope is higher than 1.25. The mentioned dependencies were obtained for random surfaces [46].

Therefore, Equation (10) relating the areal (3D) RMS slope of the equivalent sum rough surface to the RMS slopes of the individual surfaces was uncertain. However, this equation was valid in the present research. The maximum error was 0.97%, while the average error was 0.27%. The obtained finding for summation of various types of surfaces is very important.

Equation (2), describing the connection between m_4_ spectral moment and the average summit curvature, was obtained for surfaces of Gaussian ordinate distribution. In addition, the m_4_ spectral moment is the square of the mean curvature of the whole profile, not only peaks. For surfaces of non-Gaussian ordinate distributions (two-process textures, periodic surfaces), large errors of prediction of average summit curvature of the equivalent sum rough surface using Equation (11) are possible.

The difference between the average summit curvature of the sum surface and predicted by Equation (11) were higher than similar deviations concerning the Sq and Sdq parameters. In most cases, the relative errors of the Ssc parameter predictions were smaller than 8%. In two cases, the relative differences were near 12% and 11%. They corresponded to the contact of comparatively rough one-process isotropic surface (Sq = 1.4 µm, Str = 0.7) after vapor blasting with the also rough isotropic two-process surface after vapor blasting, followed by lapping (Sq = 2.8 µm, Str = 0.89), and also to the contact of one-process anisotropic cylinder surface (Sq = 2.1 µm, Str = 0.024) with two-process surface after vapor blasting and lapping (Sq = 2.5 µm, Str = 0.85). It seems that such errors were caused by the two-process character of one of the surfaces in contact. However, even the errors of 12% are comparatively small compared to errors of surface topography measurement [47]. The average relative error of the Ssc parameter prediction was 3.1%. However, the mean error of the Spc parameter expectation was 33.5%. It was probably caused by a segmentation.

The density of summits is related to spectral moments by Equation (3). However, this equation was developed by Nayak [2] for description of isotropic surfaces of Gaussian ordinate distribution. Its application for other surfaces (anisotropic, two-process, or deterministic) is questionable. For similar values of the Ssc and Sdq parameters, the density of summits is smaller for anisotropic surfaces compared to isotropic textures. The density of summits of two-process random surface is higher compared to the asperity density of one-process textures. However, there are opinions that not all the summits should be taken into consideration in the contact analysis of two-process surfaces [16,48]. Because of the difficulty of using Equation (3), areal summit density of the equivalent sum rough surface was predicted using the weighting average method—Equation (12). All summits were taken into consideration. The maximum error was 7.1%, while the average error was 3.3%. The correct results were obtained, because surfaces of various types were summed.

Prediction of the values of the Str parameter sometimes yield wrong results. The errors depend on the character of the analyzed surface. For example, when anisotropic surfaces were summed, the predicted Str parameter by weighting average method was underestimated. It this case, for similar amplitudes of two surfaces, the Str parameter of the equivalent sum surface was higher than a mean value of the Str parameter of two individual textures—Table 5 and Figure 10. A different situation occurred when isotropic surface was added to anisotropic surface. In this case, the anisotropic character of one surface decided about the Str parameter of the sum surface, and the predicted value of the Str parameter was overestimated (Table 6 and Figure 12). However, after addition of isotropic surfaces, the correct results of surface topography prediction were typically obtained (the errors were smaller than 10%). For all sum surfaces studied, the average error of the Str parameter prediction was 16.5%, while the maximum error was 90%.

A similar situation took place during analysis of the Sal parameter of the equivalent sum surface. When two surfaces of small values of the autocorrelation length were summed, frequently, the Sal parameter of the equivalent surface was higher than the value predicted using the weighted average method—Table 4. Similar to prediction of the Str parameter, when surface of small value of the Sal parameter was added to surface of higher correlation length, the Sal parameter of the sum surface was smaller than the predicted value—Table 5. However, the results were better than those obtained for the Str parameter prediction, the average error of the Sal parameter prediction was 11.2%, while the maximum error was 28.2%.

The application of the weighting average method assured good predicted values of the emptiness coefficient Sp/Sz of the equivalent sum rough surfaces. In two cases, errors were higher than 10%—the predicted values were underestimated. This situation occurred for the contact of the one-process surface with the two-process surface. These errors were considerably reduced to values smaller than 8% when the Sq parameter of the two-process surface was replaced by the Spq parameter (plateau root mean square roughness [16,48,49,50,51]). The average value of the Sp/Sz ratio prediction was 3.8%. When one-process surfaces were summed, the relative errors were smaller than 6.5%. Good results were obtained, taking into account comparative high sensitivity of the Sp/Sz parameter on measurement errors [47].

Prediction of the Sq/Sa ratio by the weighting average method assured good results. The average error of parameter prediction was 1.6% and the maximum error was 7.1%.

The errors of predicting the values of the skewness, Ssk, of equivalent rough surfaces were comparatively large. Especially, for the contact of two surfaces characterized by negative values of the Ssk parameter, the predicted value of the Ssk parameter was smaller than that of the sum surface—Table 4. A similar situation occurred for the contact of surface with symmetric ordinate distribution and the two-process surface—Table 6 and Figure 14. Large relative errors were also found for the contact of both one-process surfaces. However, they were caused by the value of the Ssk parameter close to 0. The relative error of Ssk parameter prediction was 26.2%, while the maximum error was 62%.

The relative errors of the Sku parameter prediction corresponded to large errors of the Ssk parameter forecasting, which occurred for the contact of both two-process textures (Table 4) and for the contact of the one-process surface and two-process surface (Table 6 and Figure 14). In those cases, the predicted values of the kurtosis, Sku, were overestimated. However, for the contact of two one-process textures, the errors of the Sku parameter prediction were lower than those of the Ssk parameter expectation, because the value of Sku is typically higher than the value of Ssk. The average error of the Sku parameter prediction was 13.1%, while the maximum error was 45.2%.

The question arises, how can the results of parameters’ predictions of equivalent sum surface be applied? In some cases, when the statistical models of the contact of rough surfaces are used, important parameters such as the average summit curvature, the areal density of summits, and the standard deviation of asperity heights can be predicted. In other cases, and when the deterministic models are applied, parameters’ predictions can be used in initial research. It is important that surface texture, also including texture of the equivalent sum surface, can be computer generated [52,53]. Thanks to this, the cost and time of experimental investigations can be considerably reduced. Based on predicted parameters of the sum surfaces, the plasticity index can be calculated. It describes surface ability to the plastic deformation and hence to wear. The classical version of this index developed by Greenwood and Wiliamson [54] depends on the standard deviation of summit heights and the average summits curvature. According to other works, this index is proportional to surface slope [28,29,30].

## 6. Conclusions

1. In this work, we analyzed the relationships among the parameters of two contacted surfaces on parameters of equivalent surface, for which ordinates are sums of ordinates of both surfaces. Surfaces of various types (one- and two-process, isotropic and anisotropic, random or periodic) were studied.
2. Selected parameters: Sq, Sdq, Ssc, Sds, Sp/Sz, and Sq/Sa, of sum surfaces were predicted precisely when the parameters of two individual surfaces are known. The other parameters: Sal, Str, Ssk, and Sku, were anticipated with lower accuracy.
3. The parameters Sq, Sdq, and Ssc were predicted based on the changes of profile spectral moments during surfaces’ summation. The results revealed that RMS height Sq and RMS slope Sdq were predicted with very high accuracy. The maximum errors were smaller than one percent, and the average deviations were about 0.3%. In most cases, the relative errors of the Ssc parameter prediction were smaller than 8%, while the average errors were near 3%.
4. The remaining parameters of equivalent sum surface were predicted on the base of parameters of both surfaces in contact, weighted by the values of the Sq parameter of these structures. The maximum errors of the summit density, Sds, and the Sq/Sa ratio predictions were near 7%, while the average errors were near 3% and 1.5%, respectively. In most cases, relative discrepancies between Sp/Sz parameter values of equivalent rough sum surfaces and predictions were smaller than 10% and the average errors were about 4%.
5. During parameters selection, it was found that the pair of parameters, Sp/Sz (the emptiness coefficient) and Sq/Sa, better described the shape of the ordinate distribution of diversified surface textures than the typically applied set, the skewness, Ssk, and the kurtosis, Sku.

## Figures and Tables

**Table 1:** The values of the linear correlation coefficient, r, among surface texture parameters.

Parameter	Ssk	Sku	Sq/Sa	Sp/Sz
Ssk	1			
Sku	−0.77	1		
Sq/Sa	−0.82	0.91	1	
Sp/Sz	0.71	−0.48	−0.45	1

**Table 2:** Relative errors of parameters predictions.

Parameter	Average Error, %	Maximum Error, %
Sq	0.31	0.99
Sdq	0.27	0.97
Ssc	3.1	12.1
Sds	3.5	7.1
Sp/Sz	3.8	12.3
Sq/Sa	1.6	7.1
Ssk	26.2	62.1
Sku	13.1	45.2
Str	16.5	89.9
Sal	11.3	28.2

**Table 3:** Selected parameters of surface A, of surface B, of the sum surface, and of predicted parameters of the sum surface.

Parameter	Surface A	Surface B	Sum Surface	Predicted Sum Surface	Unit
Sq	0.047	1.89	1.89	1.89	µm
Sdq	0.016	0.46	0.46	0.46	-
Ssc	7.28	139.5	139.5	139.7	1/mm
Sds	6094	3593	3612	3654	1/mm^2^
Sp/Sz	0.42	0.39	0.39	0.39	-
Sq/Sa	1.31	1.31	1.31	1.31	-
Ssk	−0.3	−0.65	−0.64	−0.64	-
Sku	3.04	4.73	4.68	4.71	-
Str	0.033	0.8	0.83	0.78	-
Sal	0.0078	0.013	0.014	0.013	mm

**Table 4:** Selected parameters of surface C, of surface D, of the sum surface, and of predicted parameters of the sum surface.

Parameter	Surface C	Surface D	Sum Surface	Predicted Sum Surface	Unit
Sq	0.7	0.79	1.05	1.05	µm
Sdq	0.13	0.16	0.21	0.21	-
Ssc	41.5	57.4	73.2	70.9	1/mm
Sds	5199	6028	5388	5639	1/mm^2^
Sp/Sz	0.2	0.31	0.26	0.26	-
Sq/Sa	1.391	1.41	1.34	1.4	-
Ssk	−2.15	−1.9	−1.44	−2.02	-
Sku	9.51	7.68	5.71	8.54	-
Str	0.028	0.019	0.29	0.023	-
Sal	0.016	0.016	0.023	0.016	mm

**Table 5:** Selected parameters of surface E, of surface F, of the sum surface, and of predicted parameters of the sum surface.

Parameter	Surface E	Surface F	Sum Surface	Predicted Sum Surface	Unit
Sq	2.06	2.9	3.57	3.56	µm
Sdq	0.23	0.53	0.58	0.58	-
Ssc	62.4	157.9	177.5	169.8	1/mm
Sds	3239	3056	3098	3132	1/mm^2^
Sp/Sz	0.56	0.44	0.47	0.49	-
Sq/Sa	1.32	1.33	1.3	1.32	-
Ssk	−0.32	−0.44	−0.3	−0.39	-
Sku	4.39	4.62	3.9	4.52	-
Str	0.024	0.86	0.62	0.51	-
Sal	0.031	0.02	0.024	0.025	mm

**Table 6:** Selected parameters of surface G, of surface H, of the sum surface, and of predicted parameters of the sum surface.

Parameter	Surface G	Surface H	Sum Surface	Predicted Sum Surface	Unit
Sq	1.37	1.26	1.86	1.86	µm
Sdq	0.38	0.19	0.42	0.42	-
Ssc	120.3	37.2	131.4	125.9	1/mm
Sds	4137	4136	3978	4136	1/mm^2^
Sp/Sz	0.48	0.143	0.36	0.32	-
Sq/Sa	1.39	1.62	1.41	1.5	-
Ssk	−0.52	−3.26	−1.23	−1.84	-
Sku	5.86	16.67	6.82	11.05	-
Str	0.72	0.85	0.79	0.78	-
Sal	0.0091	0.025	0.017	0.017	mm

## Nomenclature

Sa (Ra)	arithmetical mean height
Sal	fastest decay autocorrelation length
Sds	areal density of summits
Sdq	root mean square gradient
Sku (Rku)	kurtosis
Sp (Rp)	maximum peak height
Spc	arithmetical mean peak curvature
Spd	density of peaks
Spq	plateau root mean square roughness
Sq (Rq)	root mean square height
Ssc	average summit curvature
Ssk (Rsk)	skewness
Str	texture aspect ratio
Sv (Rv)	maximum valley depth
Sz (Rz)	maximum height
